# Secondary Folds Contribute Significantly to the Total Surface Area in the Olfactory Organ of Chondrichthyes

**DOI:** 10.3389/fphys.2019.00245

**Published:** 2019-03-12

**Authors:** Sara Ferrando, Andrea Amaroli, Lorenzo Gallus, Stefano Aicardi, Davide Di Blasi, Jørgen S. Christiansen, Marino Vacchi, Laura Ghigliotti

**Affiliations:** ^1^Department of Earth, Environmental, and Life Sciences, University of Genoa, Genoa, Italy; ^2^Department of Surgical Sciences and Integrates Diagnostics, University of Genoa, Genoa, Italy; ^3^National Research Council of Italy, IAS, Genoa, Italy; ^4^Department of Arctic and Marine Biology, UiT The Arctic University of Norway, Tromsø, Norway; ^5^Environmental and Marine Biology, Åbo Akademi University, Turku, Finland

**Keywords:** cartilaginous fish, olfactory rosette, morphology, shape analysis, histology

## Abstract

The olfactory organ of Chondrichthyes is characterized by a central support with several lamellae covered by a sensory olfactory epithelium. Although secondary folds are present on the lamellae in all the chondrichthyan species analyzed to date, their shape and size have not been described. We here analyze the olfactory organ of 13 elasmobranch and 1 holocephalan species, describe the shape of the secondary folds and evaluate how they contribute to the epithelial surface area. The secondary folds vary in shape and size, and they should always be considered when comparing the epithelial surface area among species; in fact, the increase of the area, due to the presence of the secondary folds, spans from 70 to 495% in the considered species. Because of the complexity of the shapes, we approach the description of the secondary folds by analyzing histological sections of the olfactory lamellae to obtain illustrative silhouettes. We introduce two indexes in order to describe a 2D-sectioned shape of the secondary folds. Considering the different numerical parameters which describe the morphology of the olfactory organ (secondary folds included), a principal component analysis elucidates the possible ecological role and phylogenetic relationship of the chondrichthyan olfactory organ.

## Introduction

Olfaction plays important roles in feeding (Gardiner et al., 2014), mating (e.g., Kajiura et al., 2000), and homing (Gardiner et al., 2015) of Chondrichthyes. Still, some anatomical aspects of the olfactory organ need to be elucidated. The olfactory organ of Chondrichthyes is composed by an array of olfactory lamellae attached to a central support known as the raphe. The morphology is further complex by the presence of secondary folds on both faces of each olfactory lamella (Holl, 1973; Meng and Yin, 1981a,b; Theisen et al., 1986; Savel’ev and Chernikov, 1994; Takami et al., 1994; Fishelson and Baranes, 1997; Theiss et al., 2009; Cox, 2013; Howard et al., 2013; Ferrando et al., 2016, 2017a,b). The lamellae and folds are covered by the olfactory epithelium, where the olfactory receptor neurons are located. From the olfactory receptor neurons, axons project to the telencephalon and, particularly, to the olfactory bulb, which is the primary site of olfactory signal integration (Dryer and Graziadei, 1996). Chondrichthyes share complex olfactory organs, yet they are characterized by a noteworthy morphological variability among species (e.g., Schluessel et al., 2008, 2010; Meredith and Kajiura, 2010). The number of primary olfactory lamellae does not vary ontogenetically (Theiss et al., 2009) nor with body size, but ranges between about 30 to 700 lamellae among species (Ferrando et al., 2017b). Anatomical features, such as lamellar number and lamellar surface area, have been used to investigate the relationship between form and function of the olfactory organ. For example, the lamellar number and surface area have been correlated to life history traits and habitat use, e.g., bentho-pelagic or pelagic life modes (Schluessel et al., 2008; Meredith and Kajiura, 2010).

The correlation of the size of the olfactory structures (sensory epithelium, nerve, and olfactory bulb) with the olfactory capability is a long standing problem in sensory biology of vertebrates. In mammals, neither absolute nor relative size of the olfactory bulb has been successfully related to the odor discrimination performance (e.g., Rizvanovic et al., 2013). Indeed, the assumption that differences in the size of the olfactory structures correlate with the olfactory sensitivity is not supported in teleost fish (Hansen and Zielinski, 2005). In Chondrichthyes, Meredith and Kajiura (2010) showed that the surface area of the olfactory organ in five species do not correlate with the amino acid sensitivity threshold in electrophysiological experiments. Noteworthy the presence of secondary folds is largely ignored in anatomical and physiological studies on Chondrichthyes (Kajiura et al., 2005; Schluessel et al., 2008, 2010; Meredith and Kajiura, 2010). But given that the shape and relative size of the secondary folds are species-specific and highly variable (Schluessel et al., 2008), their omission likely blurs comparative analyses of the entire epithelial surface area among chondrichthyan species. Furthermore, the shape and size of the secondary folds probably affect the water flow across the lamellae and on the olfactory epithelium. The olfactory epithelium of fishes is covered by mucus, where odorants and the specialized dendritic part of the olfactory neurons are in contact (Nevitt, 1991); the dynamics at the water/mucus interface possibly affect the molecule exchange between the two fluids.

Here we provide an overview on the secondary folds in Chondrichthyes. Although their presence is easily detectable, their actual shape is difficult to determine. The 3D-shape of the secondary folds is difficult to view both by stereomicroscope and SEM, because they are often branched, partially overlapping, and their size is beyond the resolution limit of the CT-scan used to date (e.g., Abel et al., 2010). Hence, we used histology to evaluate and describe the 2D-shape of the secondary folds. This allowed us to address previously undescribed aspects of the morphology of the olfactory organs of Chondrichthyes. Our study examines 14 chondrichthyan species and contributes to the still-weakly understood form-and-function relationships of the olfactory organ for this group of fishes.

## Materials and Methods

### Sampling

Overall 16 specimens belonging to 13 elasmobranch and 1 holocephalan species were collected for this study (Table 1). The elasmobranch species represent 10 different families and are distributed over the three super-orders Squalomorphi (6 species), Galeomorphi (3 species), and Batoidea (4 species).

**Table 1:** The specimens of Chondrichthyes used in this study represent 10 families of elasmobranch e 1 of holocephalan.

Sub-class (sc) or super-order (so)	Family	Species	Common name	Abbreviation	Sex	Size (cm)	Year of sampling	Place of sampling
sc: Holocephali	Chimaeridae	*Chimaera monstrosa*	Rabbit fish	Cm	M	52 (SL)	2013	Ligurian Sea (NW Mediterranean Sea)
so: Squalomorphi	Dalatiidae	*Dalatias licha*	Kitefin shark	Dl	F	35.5 (TL)	2018	Ligurian Sea (NW Mediterranean Sea)
so: Squalomorphi	Etmopteridae	*Etmopterus spinax*	Velvet belly	Es	F	37.5 (TL)	2013	Ligurian Sea (NW Mediterranean Sea)
so: Galeomorphi	Scyliorhinidae	*Galeus melastomus*	Blackmouth catshark	Gm1	?	40 (TL)	2017	Ligurian Sea (NW Mediterranean Sea)
so: Galeomorphi	Scyliorhinidae	*G. melastomus*	Blackmouth catshark	Gm3	F	14.5 (TL)	2017	Ligurian Sea (NW Mediterranean Sea)
so: Squalomorphi	Hexanchidae	*Heptranchias perlo^∗^*	Sharpnose sevengill shark	Hp	F	108 (TL)	2015	Ligurian Sea (NW Mediterranean Sea)
so: Galeomorphi	Carcharinidae	*Prionace glauca*	Blue shark	Pg	M	43 (TL)	2013	Ligurian Sea (NW Mediterranean Sea)
so: Batoidea	Dasyatidae	*Pteroplatytrygon violacea*	Pelagic stingray	Pv	F	107 (DW)	2015	Tyrrhenian Sea (Mediterranean Sea)
so: Batoidea	Rajidae	*Raja brachyura*	Blonde ray	Rb	F	98.5 (DW)	2015	Tyrrhenian Sea (Mediterranean Sea)
so: Batoidea	Rajidae	*Raja miraletus*	Brown ray	Rm	M	41 (DW)	2015	Tyrrhenian Sea (Mediterranean Sea)
so: Batoidea	Rajidae	*Raja polystigma*	Speckled ray	Rp	F	32 (DW)	2015	Tyrrhenian Sea (Mediterranean Sea)
so: Galeomorphi	Scyliorhinidae	*Scyliorhinus canicula*	Lesser spotted dogfish	Sc7	F	21 (TL)	2018	Ligurian Sea (NW Mediterranean Sea)
so: Galeomorphi	Scyliorhinidae	*S. canicula*	Lesser spotted dogfish	Sc8	M	35.5 (TL)	2018	Ligurian Sea (NW Mediterranean Sea)
so: Squalomorphi	Somniosidae	*Somniosus microcephalus^∗^*	Greenland shark	Sm	F	230 (TL)	2013	Kejser Franz Josephs Fjord (NE Greenland)
so: Squalomorphi	Somniosidae	*Somniosus rostratus*	Little sleeper shark	Sr	F	96 (TL)	2015	Ligurian Sea (NW Mediterranean Sea)
so: Squalomorphi	Squalidae	*Squalus blainville*	Longnose spurdog	Sb	F	53 (TL)	2015	Tyrrhenian Sea (Mediterranean Sea)

*^∗^The olfactory organs of these specimens are described in previous publications (Ferrando et al., 2016, 2017a). DW, disk width; SL, standard length; TL, total length.*

Mediterranean specimens were caught as by-catch by professional fishermen in the Ligurian Sea, North-West Mediterranean Sea, and in the Tyrrhenian Sea, close to Sardinia, and obtained for this work as donation; the specimens were not hunted for specific scientific purposes, they have come on board dead, and used only if the death was evaluated to be not occurred prior of 4 h. The Italian law “Decreto Legislativo 4 marzo 2014, n. 26,” that implemented the “European Directive 2010/63/UE,” which does not consider animal testing but the use of fish obtained as by-catch by professional fishermen, was followed.

The Greenland shark *Somniosus microcephalus* specimen was captured by scientific long lines from the RV Helmer Hanssen (Christiansen, 2012) in Northeast Greenland in August 2013. All procedures were conducted in accordance with the Animal Welfare Act and were approved by the Arctic University of Norway, Norway. The capture of sharks was carried out in strict accordance with laws and regulations and with authorization from the Government of Greenland (Ministry of Fisheries, Hunting and Agriculture, document number 935119).

The gross anatomy of olfactory organs of the sharpnose sevengill shark *Heptranchias perlo* and *S. microcephalus* has been described in two previous papers (Ferrando et al., 2016, 2017a). The olfactory organs were dissected from the specimens, fixed in 4% paraformaldehyde in 0.1 M phosphate-buffered solution (PBS, pH 7.4), washed in PBS and stored in ethanol (70% in distilled water).

### Gross Morphology

**FIGURE 1:**
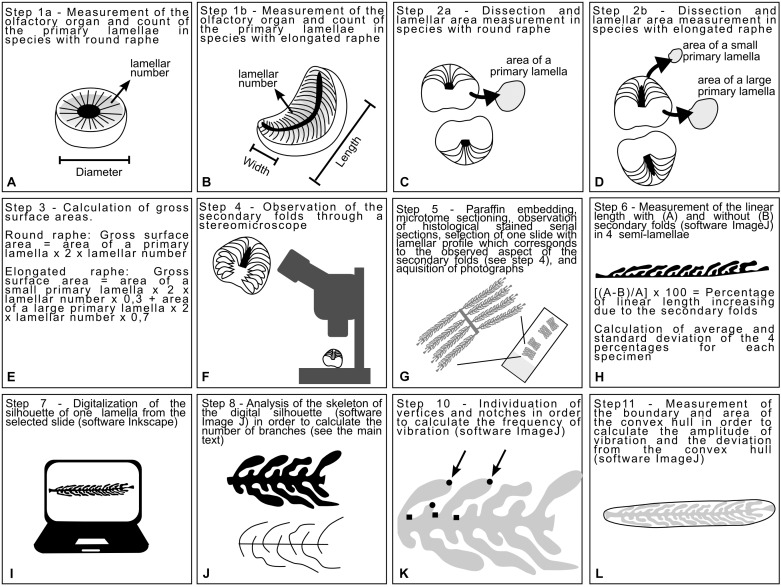
Diagrams explaining the process to obtain the measurements analyzed in this study. **(A,B)** Measures of the olfactory organ and lamellar number were acquired for both species with round and elongated raphe. **(C,D)** The olfactory organs were dissected in order to measure some lamellae. **(E)** The measure of one or two lamellae and the lamellar number were used to infer a proxy of the gross surface area of the organ. **(F)** The olfactory organs were observed through the stereomicroscope with particular attention to the 3D shape of the secondary folds. **(G)** The olfactory organ were processed with standard histological methods. **(H)** A slide were selected according to the shape of the secondary folds. The linear length and the respective boundary with secondary folds of four primary lamellae were measured and used in order to calculate the percentage of surface increase due to the secondary folds. **(I)** One of the selected primary lamellae was digitalized obtaining a silhouette for each specimen. **(J)** Each silhouette was skeletonized and the skeleton was analyzed in order to calculate the index of secondary fold branching. **(K)** The Harris corners on each silhouette were individuated by ImageJ and then vertices and notches were manually distinguished. The frequency of vibration, calculated using the number of vertices and notches were, was used in the calculation of the Brinkhoff index. **(L)** The convex hull was traced for each silhouette; its surface area and boundary length were used in the calculation of the Brinkhoff index.

All the analyses in this study were taken from the right olfactory organ, except for *H. perlo* and the speckled ray *Raja polystigma*. Two species (*H. perlo* and the rabbit fish *Chimaera monstrosa*) had round raphe surrounded by lamellae of homogeneous size and shape thus we measured the diameter of their olfactory organ (Figure 1A). Most of the other species had elongated raphe, with two arrays of lamellae along at the two sides. In this case, the length and width of their olfactory organ were measured (Figure 1B). The lamellar number (number of primary lamellae counted in a single olfactory organ) sensu Ferrando et al. (2017b), was evaluated for all the specimens (Figure 1A,B). Then the organ was dissected. In specimens with round raphe, the primary lamellae are all comparable in size; the olfactory organ was dissected in the middle, in order to photograph (with a reference scale) the face of two primary lamellae and a stripe of raphe between them (Figure 1C). Using the software ImageJ, the surface area of one face of one primary lamella, was measured, multiplied by two in order to consider the two faces, and then multiplied for the previously determined lamellar number (Figure 1E). This calculated area was considered the gross surface area that is the surface area without considering the secondary folds which are present of both the faces of each primary lamella. In specimens with elongated raphe, the primary lamellae have quite different dimensions along the raphe. They are larger in the middle part of the raphe and smaller toward the tips. The actual evaluation of the surface area would require the complete dissection of the olfactory organ, in order to measure each lamella. Especially in small olfactory organs, the dissection of the lamellae damages the tissue and limited further histological investigation. Therefore, we developed an algorithm to evaluate the gross surface area of an elongated olfactory organ, measuring only the surface area of one of the largest (middle part of the organ) and one of the smallest (peripheral part of the organ) lamella. In this way, the olfactory organ could be dissected in the middle in order to photograph one of the largest lamellae, and then in the peripheral portions, in order to photograph the face of one of the smallest lamellae (Figure 1D). The gross surface area of two species was evaluated by dissecting completely the olfactory organ. The Greenland shark *S. microcephalus*, and the blue shark, *Prionace glauca*, where chosen as examples of species with elongated raphe: *S. microcephalus* has a relatively low lamellar number and a slightly bent raphe, while *P. glauca* has a relatively high lamellar number and a quite linear raphe. In *S. microcephalus* the measured gross surface area corresponded to the surfaces area of the largest lamella surface area (considering both the faces) multiplied for the 60% of the lamellar number, plus the smallest lamella surface area multiplied for the remaining 40% of the lamellar number. In *P. glauca*, where the large lamellae occupy a wider zone of the raphe, the gross surface area corresponded to the surfaces area of the largest lamella surface area multiplied for the 80% of the lamellar number, plus the smallest lamella surface area multiplied for the remaining 20% of the lamellar number. In order to use only one formula to calculate the gross surface area of all the specimens with elongated raphe, we choose to use equal, average percentages. Thus, the gross surface area was calculated as the surfaces area of the measured largest lamella surface area (considering both the faces) multiplied for the 70% of the lamellar number (average between 60 and 80%), plus the smallest lamella surface area (considering both faces) multiplied for the 30% of the lamellar number (average between 20 and 40%). This calculated gross surface area is a proxy for the actual surface area and is likely an underestimate of the area in species that possess an elongate linear raphe and an overestimate of the area in species that possess a bent raphe.

The dissected organs were observed and photographed by a Zeiss Stemi 2000^1^ stereomicroscope equipped with a CellPad E camera (TiEsseLab S.r.l.^2^). This step allowed us to observe the morphology of the secondary folds (Figure 1F).

### Histology

Fixed olfactory organs were embedded in Paraplast (McCormick Scientific, United States) and sectioned into 5-μm thick sections according to a cutting plane which sections all the lamellae at a similar angle (Figure 1G). Histological observations were performed by Hematoxylin–eosin. Sections were examined by a Leica DMRB light microscope, and images were acquired with a Leica CCD camera DFC420C (Leica, Switzerland).

### Calculation of the Lamellar Surface Area Including Secondary Folds

Based on the observations of olfactory lamellae *in toto* (aimed at observing the 3D aspect of the secondary folds, Figure 1F), olfactory organ sections were selected for each specimen, with an illustrative 2D shape of the lamellae and their secondary folds. In different species, the lamellae can be closed or opened on the raphe and can slightly affect the cutting plane. The variability in the shape of the whole olfactory organ, the shape of the raphe (round, elongated linear, or elongated bent), and the position of the lamellae (closed or opened on the raphe) make it difficult to select the same section level along the lamellae of each specimen. Therefore sections were not acquired very close to the tip of the lamellae, nor very close to the base, but close near the level of the raphe.

For each specimen, the increase of the lamellar surface area due to the presence of the secondary folds was inferred from the measurement of the linear length of four lamellae and their semi-boundary, considering the secondary folds. The significance of the increase of the epithelial length due to the presence of secondary folds was tested for each specimens using an ANOVA. Then, the percentage of increase was calculated comparing the linear length of the primary lamella and its semi-boundary (Figure 1H). The percentage was calculated four times on different lamellae for each specimen, and the average used to estimate the surface area that includes the secondary folds based on the previously evaluated gross surface area. The ANOVA test and the Tukey *post hoc* test were used to test for differences among average percentage of surface area increase due to the secondary folds. For comparison of the gross and secondary fold surface areas among specimens of different sizes, we also calculated relative surface area (area/size^2^) as the ratio of area to the respective square of the disk width (for batoid specimens) and the total body length (for all other specimens).

### Fine Morphology

The histological slides used to measure the increase of the lamellar surface area (see section “Calculation of the Lamellar Surface Area Including Secondary Folds”) were also used in order to chose and draw an illustrative 2D silhouette of one lamella with secondary folds for each specimen. The silhouette of each selected primary lamellae bearing secondary folds were drawn (Figure 1I), maintaining the right proportion, tracing the histological photographs using Inkscape: Open Source Scalable Vector Graphics Editor. The silhouettes of the lamella, illustrative for each specimen, were plotted on a phylogenetic tree built online on the Interactive Tree of Life v.4 (Letunic and Bork, 2016), and based on the phylogeny of Naylor et al. (2012). Three species belong to genus *Raja* (Table 1); only one of them, the brown ray *Raja miraletus*, is present in the phylogeny of Naylor et al. (2012) and for this reason the three *Raja* species are in polytomy in the tree. The longnose spurdog *Squalus blainville* is not present in the phylogeny of Naylor et al. (2012), while there are several other *Squalus* species all grouped in the family Squalidae: the position in the tree of *S. blainville* was obtained from the position of these congeneric species. All the other species here considered are present in the phylogeny of Naylor and colleagues. The Newick format of the tree is: [*Chimaera_monstrosa* (((*Raja_brachyura*, *Raja_polystigma*, *Raja_miraletus*), *Pteroplatytrygon_violacea*) ((*Heptranchias_perlo*, (*Squalus_blainville*, (*Dalatias_licha*, (*Etmopterus_spinax*, (*Somniosus_rostratus*, *Somniosus_microcephalus*)))))) ((*Scyliorhinus_canicula*, (*Galeus_melastomus*, *Prionace_glauca*))))].

### 2D Shape Analysis

In order to obtain numerical indexes which can describe the shape of the silhouette of the selected primary lamellae and secondary folds, the skeleton analysis was performed, and the index of polygonal complexity was calculated according Brinkhoff et al. (1995). The skeleton analysis was performed using the version of ImageJ named Fiji (Schindelin et al., 2012), and it was used in order to obtain an index descriptive for the secondary fold branching. Each silhouette was skeletonized by the software (Figure 1J) and then the number of branches were automatically evaluated. The obtained number of branches depended on the complexity of the secondary folds and on their number. The number of secondary folds, in turn, depended on the size of the primary lamella and on the density of secondary folds. The secondary folds branching index was obtained by normalizing the number of branches for the number of secondary folds.

The index of Brinkhoff considers three sub-indexes: the frequency of the vibration, the amplitude of the vibration, the deviation from the convex hull. The vibration is the change of direction in the boundary of the 2D shape. The calculation of frequency of the vibration requests the count of vertices and notches of the figure; this count was performed individuating the Harris corners (by an algorithm) in the lamellae silhouettes using the OrientationJ plug-in for ImageJ (Schneider et al., 2012; Püspöki et al., 2016) and then, manually counting the notches among the Harris corners automatically individuated (Figure 1K). The frequency of the vibration spans from values close to 0 (low frequency) to values close to 1 (high frequency) and it is obtained as: 16 × (normalized number of notches – 0.5)^4^–8 × (normalized number of notches – 0.5)^2^ + 1. The “normalized number of notches” is the number of notches divided for the (number of vertices – three).

For each silhouette, a convex hull was drawn using the shape analysis plug-in for ImageJ (Figure 1L); the boundary and area of each silhouette and its convex hull were measured using ImageJ. The amplitude of the vibration was calculated as: (boundary of the silhouette – boundary of the convex hull)/boundary of the silhouette; if the silhouette were convex the amplitude of the vibration will be 0, otherwise it will tend to 1 as a unreachable limit. The deviation from the convex hull was calculated as: (area of the convex hull – area of the silhouette)/area of the convex hull; if the silhouette were convex the deviation from the convex hull will be 0, otherwise it will tend to 1 as a unreachable limit. According to Brinkhoff et al. (1995), the index of Brinkhoff is calculated as: 0.8 × amplitude of the vibration × frequency of the vibration + 0.2 × deviation from the convex hull.

### Principal Component Analysis (PCA)

A dataset of numerical measurements and indexes was used for the multivariate principal component analysis (PCA); the selected data (Table 2) are descriptive of the shape of the olfactory organ. The gross surface area and the surface area with secondary folds were not included in this analysis because of the bias in their evaluation, due to the chosen algorithm. We ran a phylogenetic PCA (pPCA, function phylo.PCA of the phytools R-package; Revell, 2012), which considers the phylogenetic relationship among specimens. The procedure uses a matrix derived from the phylogenetic tree (Revell, 2009); the Newick tree used to perform the pPCA analysis was obtained as previous indicated (see above). The length of each branch of the tree was set as 1. For the species for which we had two specimens, the lesser spotted dogfish *Scyliorhinus canicula* and the blackmouth catshark *Galeus melastomus*, only the largest specimen was considered in the pPCA analysis. The information about diet and habitat was collected for the respective species from the literature, as reported in Table 3.

**Table 2:** Measures and indexes regarding the olfactory organs.

Specimen (see Table 1)	Size (cm)	Olf. organ width (mm)	Olf. organ length (mm)	Largest lamella surface area (mm^2^)	100 ^∗^ Largest lamella surface area/size^2^	Lamellar number	Gross surface area (mm^2^)	100 ^∗^ Gross surface area/size^2^	Surface area with secondary folds (mm^2^)	100 ^∗^ Surface area with secondary folds/size^2^	Average percentage of surface area increase due to secondary folds	Standard deviation of the percentage of surface area increase due to secondary folds	Secondary folds branching index	Frequency of vibration	Amplitude of vibration	Deviation from a convex hull	Brinkhoff index
Cm	L 52	10	10	12.5	**0.46**	**34**	850	31	1819	**67**	**114**	8.5	**1.96**	0.9	0.5	0.53	**0.46**
Dl	L 35.5	7	9	7.5	**0.60**	**44**	543	43	977	**78**	**80**	10.2	**1.61**	0.61	0.42	0.3	**0.27**
Es	L 37.5	6	9	8	**0.57**	**52**	720	51	2246	**160**	**212**	39	**2.16**	0.98	0.66	0.45	**0.61**
Gm1	L 40	10	14	14	**0.88**	**38**	973	61	3240	**203**	**233**	57	**2.13**	1	0.77	0.4	**0.69**
Gm3	L 14.5	5	7	5.5	**2.62**	**40**	351	167	1755	**835**	**400**	31	**2.2**	1	0.67	0.34	**0.6**
Hp	L 108	13	13	33.5	**0.29**	**34**	2278	20	12848	**110**	**464**	38	**3.29**	0.95	0.8	0.52	**0.72**
Pg	L 43	8	13	16.5	**0.89**	**95**	2825	153	7821	**423**	**177**	9	**2.04**	0.96	0.63	0.42	**0.56**
Pv	W 107	13	26	22	**0.19**	**110**	4340	38	7378	**64**	**70**	12	**1.94**	0.92	0.39	0.51	**0.38**
Rb	W 98.5	14	28	56.2	**0.58**	**61**	6300	65	19971	**206**	**217**	30	**3.67**	1	0.67	0.37	**0.61**
Rm	W 41	10	22	17	**1.01**	**46**	1364	81	5006	**298**	**267**	29	**3.42**	0.89	0.72	0.39	**0.59**
Rp	W 32	9	15	15.1	**1.47**	**40**	1056	103	4425	**432**	**319**	32	**3.52**	1	0.75	0.31	**0.66**
Sb	L 53	7	12	14.1	**0.50**	**47**	1177	42	3872	**138**	**229**	21	**2.03**	0.94	0.68	0.49	**0.62**
Sc7	L 21	6	8	7.6	**1.72**	**34**	453	103	1586	**360**	**250**	37	**2.09**	0.87	0.69	0.28	**0.54**
Sc8	L 35.5	8	12	13	**1.03**	**34**	730	58	3249	**258**	**345**	53	**1.99**	0.98	0.75	0.4	**0.67**
Sm	L 230	35	50	220	**0.42**	**44**	11700	22	62478	**118**	**434**	93	**6.92**	0.99	0.8	0.36	**0.7**
Sr	L 96	14	23	44	**0.48**	**36**	2200	24	13090	**142**	**495**	35	**7.9**	1	0.82	0.46	**0.74**

*Data in bold have been used in order to run PCA. L, total length; W, disk width.*

**Table 3:** Ecological character of included species.

Feeding preferences				
**Species**	**Habitat**	**Teleost**	**Mollusc**	**Crustacean**	**Chondrichthyan**	**Echinoderm**	**Reference of feeding preferences**
*C. monstrosa*	Bathydemersal			20%		70%	MacPherson, 1980
*D. licha*	Bathydemersal	40%	15%	20%	25%		Barría et al., 2015
*E. spinax*	Bathydemersal	85%		15%			Fanelli et al., 2009
*G. melastomus*	Bathydemersal	20%	40%	40%			Fanelli et al., 2009
*H. perlo*	Bathydemersal	75%	20%	5%			Braccini, 2008
*P. glauca*	Pelagic-oceanic	60%	40%				Barría et al., 2015
*P. violacea*	Pelagic-oceanic	95%	5%				Lipej et al., 2013
*R. brachyura*	Demersal	95%	5%				Follesa et al., 2010
*R. miraletus*	Demersal	2%	2%	95%			Follesa et al., 2010
*R. polystigma*	Demersal	10%		80%			Barría et al., 2015
*S. canicula*	Demersal	12%	6%	75%			Valls et al., 2011
*S. microcephalus*	Benthopelagic	65%	20%		5%		Nielsen et al., 2014
*Somniosus rostratus*	Bathydemersale		95%	5%			Barría et al., 2015
*S. blainville*	Demersal	20%	11%	52%			Martinho et al., 2012

*Some minor taxonomic groups of prey are omitted, for this reason not all the sums of feeding preferences give the 100%. The habitat is from Pollerspöck and Straube (2018).*

## Results

### Gross Morphology

**FIGURE 2:**
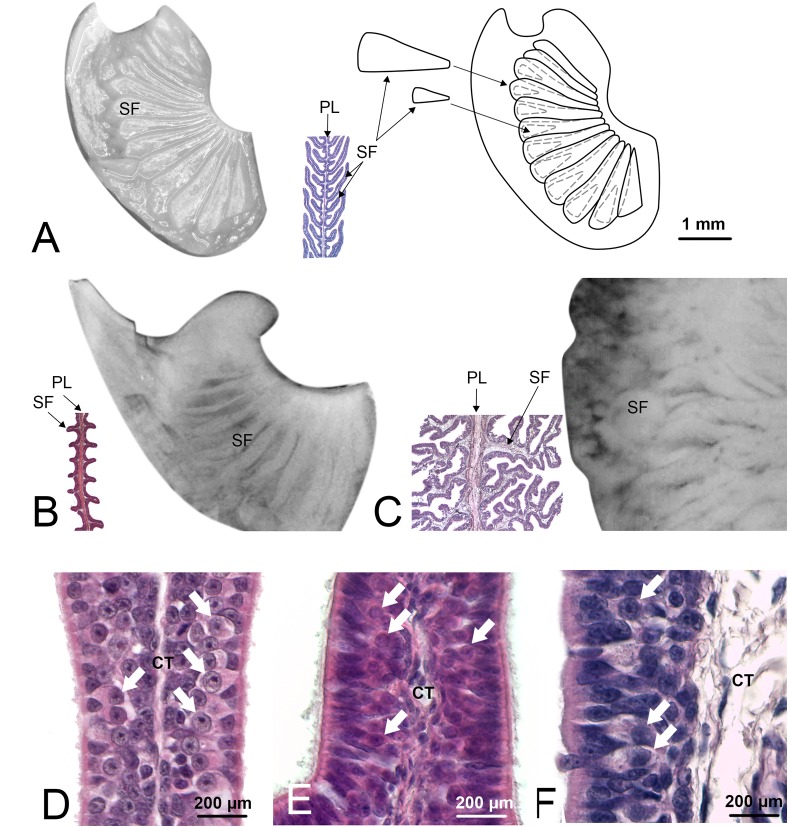
Olfactory lamellae of *Scyliorhinus canicula* (specimen Sc8), *Pteroplatytrygon violacea* (Pv), and *Somniosus microcephalus* (Sm). These three species were chosen because represent three different types of secondary folds, respectively, not branched and long so that they appear partially overlapped, not branched and short, and branched. **(A)** *S. canicula*, one of the primary olfactory lamellae was photographed through the stereomicroscope (left). Not all the secondary folds are visible. Further dissection of the lamella (not shown) allowed to draw the scheme on the right, showing also the hidden secondary folds. Between the photograph and the scheme, a partial histological section of a lamella from the same specimen, with the same magnification, is showed. The cutting plane of the histological section allows to see the secondary folds at the two sides of the primary lamella. **(B)** *P. violacea*, one of the primary olfactory lamellae was photographed through the stereomicroscope; the secondary folds are visible on the surface although it is not easy to determine the extent of these structures. The histological section helps to understand shape and size of the secondary folds. **(C)** *S. microcephalus*, one of the primary olfactory lamellae was photographed through the stereomicroscope; in order to maintain the same scale with the figures **(A,B)**, only a zone of the lamella is visible in the photograph. The secondary folds have a confused aspect through the stereomicroscope and the histological section is essential to resolve their shape, albeit in a 2D image. **(A–C)** figures, for whole mount photos, schemes, and histological sections, have the same scale bar, showed in Figure 1A. **(D–F)** Histological sections of the olfactory organ of, respectively, *S. canicula*, *P. violacea*, and *S. microcephalus.* At the two side of the connective tissue, the sensory epithelium is visible; olfactory sensory neurons can be observed (arrows) as their round nuclei with evident nucleolus are localized in the middle part of the epithelium. CT, connective tissue; PL, primary lamella; SF, secondary fold.

The olfactory organ size, as well as the surface area of the largest lamellae in the mid part of each olfactory organ were measured according to Figure 1C,D and are reported in Table 2, together with the lamellar number (Ferrando et al., 2017b), and the corresponding gross surface area (see Materials and Methods). A precise method to calculate the gross surface area of a specimen without a complete dissection of the organ is out of the aim of this study, and it would require several observations for each organ shape. Then, the gross surface area and, consequently, all the parameters based on it, are not meant to quantitatively compare the species here analyzed. They indicate the order of magnitude of the surface area in these specimens and, indeed, they suggest large differences among different species. The observation through the stereomicroscope allowed to see the shape of the secondary folds (Figure 2A–C).

### Fine Morphology

**FIGURE 3:**
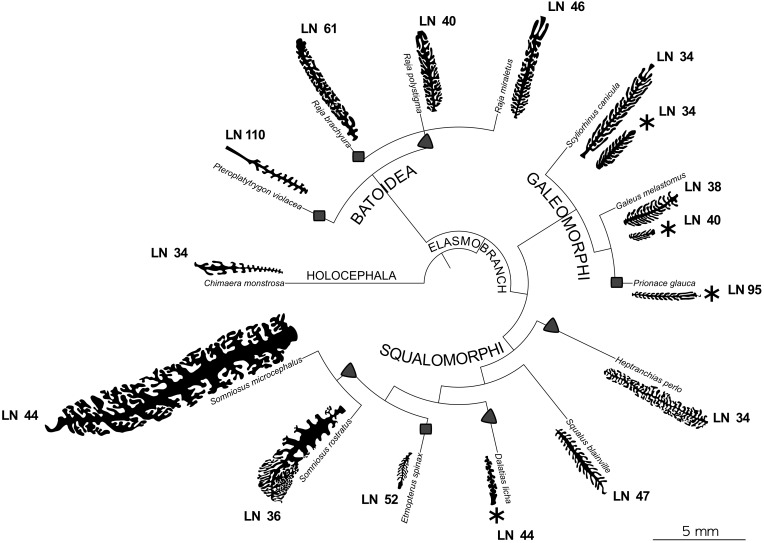
Phylogenetic tree of the species of interest. For each species the silhouette of a illustrative primary lamella with secondary folds is plotted. All the silhouettes are proportionally drawn, having the same scale. For the species where two specimens were analyzed, both the silhouettes are present. For each species the lamellar number (LN) is reported. Specimens with an asterisk are those which are possibly newborn or very young. *Chimaera monstrosa* is the outgroup and is characterized by less than 50 primary lamellae and by short, unbranched secondary folds. Squares and triangles highlight, respectively, the appearance of a number of primary lamellae higher than 49 and the appearance of branched secondary folds, considering these tracts as derived.

The epithelial length increase due to the presence of the secondary folds is significant (ANOVA *p*-value < 0.001) for each specimen. The average percentage of surface area increase due to secondary folds is variable among the specimens (Table 2), ranging from 70 to 495% (see Table 4 for the significance of the difference among the specimens). The silhouettes of primary lamellae with secondary folds illustrative of each specimens, were matched to the phylogenetic tree built according to Naylor et al. (2012), together with the lamellar number (Figure 3). The sensory epithelium, which covers the primary lamellae and the secondary folds, was similar to that already described for some of these and other species (e.g., Ferrando et al., 2010, 2016, 2017a; Figure 2D–F). The non-sensory epithelium covered the edge of the primary lamellae, as also described by Schluessel et al. (2010), while the sensory epithelium was observed, in all the specimens, to cover the face of the primary lamellae, including the secondary folds.

**Table 4:** Significance of the difference of the average percentages of increase of the lamellar surface area due to the secondary folds according to the Tukey *post hoc* test.

	Cm	Pv	Rb	Rp	Rm	Hp	Sb	Dl	Es	Sr	Sm	Sc7	Sc8	Gm3	Gm1	Pg
Cm			^∗^	^∗∗∗∗∗^	^∗∗∗^	^∗∗∗∗∗^	^∗^		.	^∗∗∗∗∗^	^∗∗∗∗∗^	^∗∗^	^∗∗∗∗∗^	^∗∗^	^∗∗∗∗∗^	
Pv			^∗∗∗^	^∗∗∗∗∗^	^∗∗∗∗∗^	^∗∗∗∗∗^	^∗∗∗∗^		^∗∗∗^	^∗∗∗∗∗^	^∗∗∗∗∗^	^∗∗∗∗∗^	^∗∗∗∗∗^	^∗∗∗∗^	^∗∗∗∗∗^	^∗^
Rb	^∗^	^∗∗∗^		^∗^		^∗∗∗∗∗^		^∗∗^		^∗∗∗∗∗^	^∗∗∗∗∗^		^∗∗^		^∗∗∗∗∗^	
Rp	^∗∗∗∗∗^	^∗∗∗∗∗^	^∗^			^∗∗∗^		^∗∗∗∗∗^	^∗^	^∗∗∗∗∗^	^∗^					^∗∗∗^
Rm	^∗∗∗^	^∗∗∗∗∗^				^∗∗∗∗∗^		^∗∗∗∗∗^		^∗∗∗∗∗^	^∗∗∗∗^				^∗∗^	
Hp	^∗∗∗∗∗^	^∗∗∗∗∗^	^∗∗∗∗∗^	^∗∗∗^	^∗∗∗∗∗^		^∗∗∗∗∗^	^∗∗∗∗∗^	^∗∗∗∗∗^			^∗∗∗∗∗^	^∗∗^	^∗∗∗∗∗^		^∗∗∗∗∗^
Sb	^∗^	^∗∗∗∗^				^∗∗∗∗∗^		^∗∗∗^		^∗∗∗∗∗^	^∗∗∗∗∗^		^∗^		^∗∗∗∗^	
Dl			^∗∗^	^∗∗∗∗∗^	^∗∗∗∗∗^	^∗∗∗∗∗^	^∗∗∗^		^∗∗^	^∗∗∗∗∗^	^∗∗∗∗∗^	^∗∗∗∗^	^∗∗∗∗∗^	^∗∗∗^	^∗∗∗∗∗^	.
Es	.	^∗∗∗^		^∗^		^∗∗∗∗∗^		^∗∗^		^∗∗∗∗∗^	^∗∗∗∗∗^		^∗∗^		^∗∗∗∗∗^	
Sr	^∗∗∗∗∗^	^∗∗∗∗∗^	^∗∗∗∗∗^	^∗∗∗∗∗^	^∗∗∗∗∗^		^∗∗∗∗∗^	^∗∗∗∗∗^	^∗∗∗∗∗^			^∗∗∗∗∗^	^∗∗∗^	^∗∗∗∗∗^	.	^∗∗∗∗∗^
Sm	^∗∗∗∗∗^	^∗∗∗∗∗^	^∗∗∗∗∗^	^∗^	^∗∗∗∗^		^∗∗∗∗∗^	^∗∗∗∗∗^	^∗∗∗∗∗^			^∗∗∗∗∗^		^∗∗∗∗∗^		^∗∗∗∗∗^
Sc7	^∗∗^	^∗∗∗∗∗^				^∗∗∗∗∗^		^∗∗∗∗^		^∗∗∗∗∗^	^∗∗∗∗∗^		.		^∗∗∗^	
Sc8	^∗∗∗∗∗^	^∗∗∗∗∗^	^∗∗^			^∗∗^	^∗^	^∗∗∗∗∗^	^∗∗^	^∗∗∗^		.		^∗^		^∗∗∗∗^
Gm3	^∗∗^	^∗∗∗∗^				^∗∗∗∗∗^		^∗∗∗^		^∗∗∗∗∗^	^∗∗∗∗∗^		^∗^		^∗∗∗∗^	
Gm1	^∗∗∗∗∗^	^∗∗∗∗∗^	^∗∗∗∗∗^		^∗∗^		^∗∗∗∗^	^∗∗∗∗∗^	^∗∗∗∗∗^	.		^∗∗∗^		^∗∗∗∗^		^∗∗∗∗∗^
Pg		^∗^		^∗∗∗^		^∗∗∗∗∗^		.		^∗∗∗∗∗^	^∗∗∗∗∗^		^∗∗∗∗^		^∗∗∗∗∗^	

*See the table for the values of the average percentage. Empty: p-value ≥ 0.1; (.): 0.05 ≤p-value < 0.1; (^∗^):0.01 ≤p-value < 0.05; (^∗∗^): 0.001 ≤p-value < 0.01; (^∗∗∗^): 0.0001 ≤p-value < 0.001; (^∗∗∗∗^): 0.00001 ≤p-value < 0.0001; (^∗∗∗∗∗^) p-value < 0.000001. The acronyms of the specimens are in Table 1.*

### Calculation of the Lamellar Surface Area Including Secondary Folds

**FIGURE 4:**
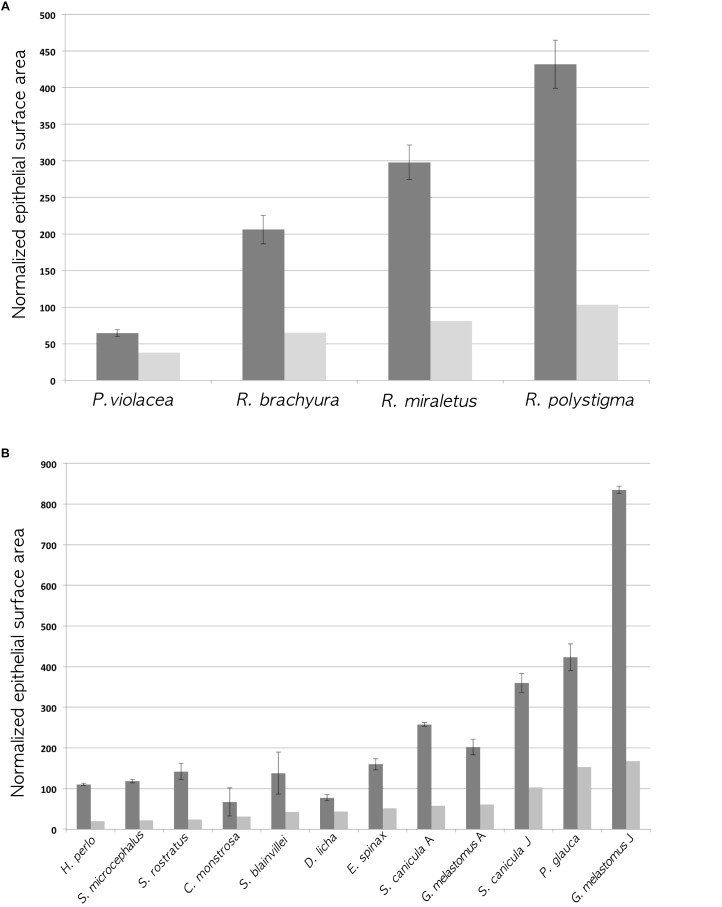
Histograms of the normalized gross surface area (light gray; see Table 2 ninth column for values) and the normalized surface area with secondary folds (dark gray; see Table 2 eleventh column for values). The error bars of the latter are calculated using the standard deviation of the percentage of increase of the surface (see Table 2 twelfth column for the standard deviations). **(A)** Batoidea specimens. The surface area (mm^2^) multiplied by 100, was normalized dividing for the square disk width (cm^2^). **(B)** Non-batoidea specimens. The surface area (mm^2^) multiplied by 100, was normalized dividing for the square total length (cm^2^).

The calculated proxy for the lamellar surface area, with and without considering the secondary folds, shows that, obviously, the surface area with the secondary folds/size^2^ is always larger than the gross surface area/size^2^, where “size” could be alternatively the total length or the disk width as indicated before and in Table 2; because the percentage of increase of the lamellar surface area due to the secondary folds was not the same between specimens, those with the largest gross surface area/size^2^ did not have always the largest surface area with the secondary folds/size^2^. The surface area with the secondary folds/size^2^ represents the proxy for the calculated whole surface area of one olfactory organ normalized for the specimen size and should be very important for comparing the olfactory organ morphology among different species. As it is secondary-folds informed, it should be more significant than the normalized gross surface area, which has been used in the literature to date (Schluessel et al., 2008; Meredith and Kajiura, 2010). The two normalized areas, gross surface and surface with secondary folds, for each specimen, are compared in Figure 4. It is possible to observe that, although the gross surface area is a proxy of the real one, the presence of secondary folds is likely to completely alter the comparisons among different specimens.

### 2D Shape Analysis

The frequency of the vibration, amplitude of the vibration, the deviation from the convex hull, the Brinkhoff index and the secondary fold branching index regarding the silhouettes of the illustrative primary lamellae and secondary folds are reported in Table 2. The Brinkhoff index put together the frequency of the vibration, the amplitude of the vibration and the deviation from the convex hull. Note that the Brinkhoff index describes the complexity of the silhouettes (2D), not that of the primary lamellae with secondary folds, which are 3D objects. The secondary fold branching index analyzes the branching of the secondary folds in the selected cutting plane and do not take in account other zones of the lamella. The Brinkhoff index does not correlate with the secondary fold branching index, nor with the normalized surface area. The normalized surface area with does not correlate with the secondary fold branching index.

The secondary fold branching index divides the specimens in three groups: those with a value close to 2 (the secondary folds are mainly not branched), close to 3–4 (most of the secondary folds have two or a few branches), and close to 7 (the secondary folds are very branched).

**FIGURE 5:**
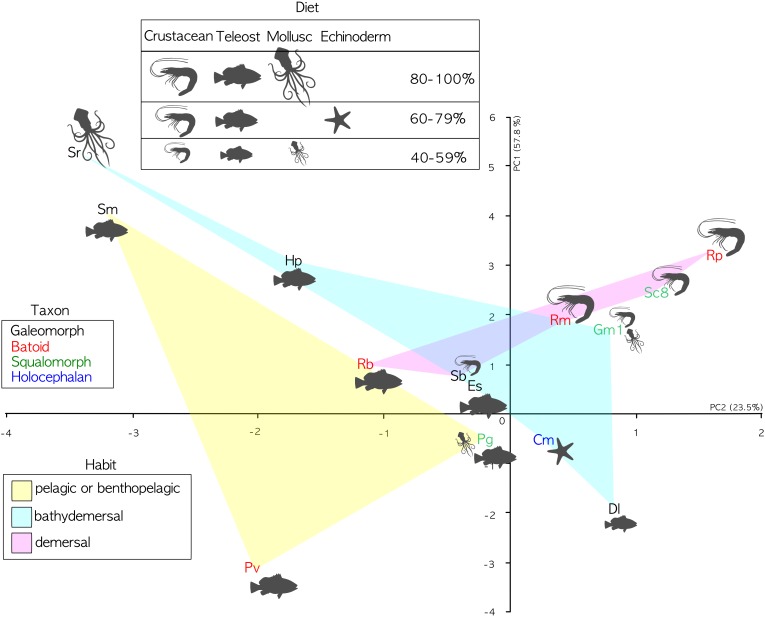
PCA analyses. PC1 explains 57.8 of variance; PC2 explains 23.5 of variance. Specimen abbreviations are written in different color according to sub-class or super-order: blue denotes the Holocephali, red Batoidea, green Galeomorphi, and black Squalomorphi. The light colored areas represent different habitats: yellow pelagic and benthopelagic; pink demersal; and light cyan bathy-demersal. Feeding preferences (cf. Table 3) are plotted only when a given prey item represents a substantial part of the diet (>40%). The larger the drawing of the prey item, the higher the percentage.

The phylogenetic informed PCA was related to the dataset in Table 2, considering only the bold columns. We retained the two first axes of the pPCA which explain 81.3% of the total variation. Loadings of the first principal component (PC1) and second principal component (PC2) are found in Table 5. The PC1 positively correlates with the calculated percentage of increase of the lamellar surface area due to the secondary folds, with the index of secondary folds branching and with the Brinkhoff index; it negatively correlates with the lamellar number. The PC2 positively correlates with the size of the largest lamella normalized for the square of the fish size and negatively correlates with the index of secondary folds branching. On the basis of the considered data, the pelagic and bentho-pelagic species are separate from the demersal and bathy-demersal ones, which in turns share the morphospace. Some clustering is evident also regarding the diet, with species that fed mainly on fish or crustacean grouped in the left and right parts of the plane, respectively. Noteworthy, the blonde ray *Raja brachyura*, which is a demersal species but which, differently from the analyzed congeneric species, fed on fish, protrudes toward the left part of the plane (Figure 5).

**Table 5:** Principal component analysis, loadings of the first principal component (PC1) and second principal component (PC2).

	PC1	PC2
100^∗^Largest lamella surface area/size^2^	0.4565075	0.6648514
Lamellar number	-0.7052190	-0.1243288
Percentage of surface area increase due to secondary folds	0.9454380	-0.1908870
Index of secondary fold branching	0.7088766	-0.5531881
Index of Brinkhoff	0.8886499	-0.1247390

## Discussion

To the best of our knowledge, this is the first work which analyzes the secondary folds that are present along the primary lamellae in the olfactory organ of Chondrichthyes. The secondary olfactory folds range between species from very simple and short, to highly complex and branched. Although the surface areas estimated in this paper, are just proxies of the real ones, they provide an improvement over previous studies, all of which did not consider the secondary folds. The inclusion of the secondary folds significantly increases the linear length of the epithelium in the histological sections of the olfactory lamellae, therefore it substantially changes the total olfactory surface area and ultimately affects comparative analyses of Chondrichthyes. In Figure 4A, the Batoidea species do not change their rank (the species with a smaller normalized gross surface area has also the smaller area with secondary folds) but the differences among species are enhanced by the presence of secondary folds because of their variable morphology in different species. In Figure 4B, the non-batoidea specimens, which are put in order of increasing normalized gross surface area, ranked differently considering the normalized gross surface area or the normalized surface area with secondary folds. This supports the use, in future works, of the surface area with secondary folds in comparative analyses.

We introduced two descriptive indexes for the secondary-fold branching and the shape complexity as numeric proxies for the shape of the secondary folds. The two indexes describe different characters of the shape and are not correlated. The lack of correlation between the two indexes shows that they describe different features of the silhouettes, and possibly, of the secondary fold morphology. Neither index was correlated with the normalized surface area with secondary folds and this seems to confute the intuitive idea that more complex olfactory lamellae are justified by the need of a greater surface area. Also phylogenetic considerations should be cautiously done regarding the secondary fold branching index. Only two species have a secondary fold branching index close to 7, and they both belong to the genus *Somniosus*. Three of the four batoid species have a secondary fold branching index close to 3–4, and they all belong to the genus *Raja*. On the other hand, the secondary fold branching index seems unrelated to the ecology of the species or, at least, not to the ecological features here consider (habitat and feeding preference). For example, *S. microcephalus* is bentho-pelagic and as adults has a generalist diet based on fishes and marine mammals, while the little sleeper shark *S. rostratus* is bathydemersal and has a specialist diet based on cephalopods (see Table 3).

The Brinkhoff index is difficult to interpret; in fact it is not related to the shape of the secondary folds themselves, but it seems to describe in the same way large highly branched secondary folds and numerous thin not-branched secondary folds. One *G. melastomus* (Gm1), for example, has dense unbranched secondary folds and a Brinkhoff index similar to that of *S. microcephalus*, which has very branched secondary folds. Furthermore, there are large differences in the Brinkhoff index between the small and the large specimens of *S. canicula* and *G. melastomus*. The density of branches or the density of unbranched secondary folds, described by the Brinkhoff index, could be of interest with respect to the water flow and for the interaction across the water-epithelial surface. Indeed, it is widely recognized that the primary olfactory lamellae are important for the water flow within the olfactory chamber (e.g., Abel et al., 2010), thus it is reasonable that the secondary folds can in turn deeply affect the water flow over the olfactory epithelium. On the other hand, also the thickness of the secondary folds, affecting their density, is described by the Brinkhoff index, and could be related to the abundance of fila olfactoria, i.e., the axons of the epithelial sensory neuron which are running in the connective tissue in the mid-part of each lamella, toward the olfactory bulb. In this case thinner folds would indicate a lower sensory neuron abundance in the sensory epithelium. The neuron density and abundance in the epithelium and its functional role among Chondrichthyes (and also during ontogeny) deserve a deeper analysis that is beyond the scope of this paper.

Despite the relatively low number of species here considered, the phylogenetic tree, based on Naylor et al. (2012), encourages further analysis of the morphological characters of the olfactory organ. Assuming that the plesiomorphic trait is <50 lamellae and unbranched secondary folds (as in the holocephalan *C. monstrosa* and some of the elasmobranch species, see Figure 3), the increase in the number of lamellae seems to be a trait which evolved in each of the elasmobranch super-orders. The four species which have a lamellar number >50 (the pelagic stingray *Pteroplatytrygon violacea*, *R. brachyura*, the blue shark *P. glauca*, and *E. spinax*) do not share the same habitat, while they all feed on bony fishes. It is noteworthy that the piscivore *R. brachyura* has more lamellae than the other two *Raja* species (about +20), which mainly prey crustaceans. We should note that the body size of *R. brachyura* is larger than the other two *Raja* species, albeit the lamellar number is usually not correlated to body size (Theiss et al., 2009), and this lack of correlation is evident also in the species analyzed in the present work. In this frame, a higher lamellar number could be related to a long-distance chemoreception.

The branched secondary folds are present in batoids (at least in Rajidae) and in the squalomorph group, while they lacks in galeomorphs. But again, it should be stressed that only three galeomorph species were considered in this study. Three of four species with a lamellar number >50 have unbranched secondary folds, allowing to speculate that branched secondary folds and increased lamellar number could be two different strategies to increase the olfactory surface area. This could explain why secondary fold branching index and surface area seem uncorrelated if all the specimens are considered together. Indeed, *R. brachyura* adopts both the strategies, while *C. monstrosa*, *S. canicula*, *G. melastomus*, and *S. blainville* adopt none of them, although *G. melastomus* has noteworthy thin, long, unbranched, and densely packed secondary folds.

In conclusion, our examination of the chondrichthyan olfactory organ reveals that the secondary folds on the primary lamellae vary significantly in both shape and size. The secondary folds certainly affect the sensory surface area, an important, but controversial, feature for the olfactory capability in vertebrates, and should not be ignored in future works attempting to correlate olfactory anatomy to function. Despite the relatively low number of species and specimens here analyzed, it appears clear that both phylogeny and ecology influence the morphology of the olfactory organ in Chondrichthyes. Further studies, including a larger number of species and ontogenetic stages, are called for to solve form-and-function in an ecological and evolutionary context.

## Data Availability

All datasets generated for this study are included in the manuscript and/or the supplementary files.

## Author Contributions

SF, AA, LGa, and LGh conceived and designed the analysis. SF and SA collected the data. DDB, JC, and MV contributed to analysis tool and specimens. SF, AA, LGa, SA, and LGh performed the analysis. SF wrote the manuscript. AA, LGa, LGh, JC, MV, and SA improved the manuscript with constructive criticisms.

## Conflict of Interest Statement

The authors declare that the research was conducted in the absence of any commercial or financial relationships that could be construed as a potential conflict of interest.

## Footnotes

1 www.zeiss.com

2 www.tiesselab.com
